# Dielectric and mechanical properties of polyaniline-grafted sepiolite loaded PVC nanocomposites

**DOI:** 10.55730/1300-0527.3744

**Published:** 2025-03-12

**Authors:** Asif RAZA, Tariq YASIN, Muhammad NADEEM, Muhammad Arshad FARHAN

**Affiliations:** 1 Department of Chemical Engineering, Pakistan Institute of Engineering and Applied Sciences (PIEAS), Islamabad, Pakistan; 2 Division of Physics, Directorate of Science, Pakistan Institute of Nuclear Science and Technology (PINSTECH), Islamabad, Pakistan

**Keywords:** Nanohybrid polymer composite, conducting polymer, dielectric, impedance

## Abstract

Conducting polymers exhibit interesting dielectric properties due to their unique conjugated π-electron backbone. The objective of this study was to examine the dielectric properties of nanocomposites containing a conducting nanohybrid (NH) as a filler. Polyaniline was grafted onto sepiolite by in situ emulsion graft polymerization to synthesize a conducting NH. This NH was blended with poly(vinyl chloride) (PVC) using a melt blending technique. Nanocomposites with variable amounts of nanofiller were prepared and characterized. Fourier-transform infrared (FT-IR) spectra of the NH composites show strong interactions between their individual components. The addition of the nanofiller into PVC increased its thermomechanical properties. Dielectric loss factor and permittivity increased with nanofiller content and decreased frequency, exhibiting strong interfacial polarization in the low frequency range. Incorporation of the nanofiller into the PVC matrix increased conductivity up to 7.9 × 10^−7^ S/cm and substantially decreased impedance. The results demonstrate the potential of the nanocomposite for electromagnetic interference shielding, biomedical applications, biosensing, and environmental sensing.

## 1. Introduction

Conducting polymers have unique electrical and dielectric properties that can be used in scientific research and technological applications [1]. Polyaniline (PANI) has been the subject of considerable scientific research due to properties such as ease of preparation, low cost, and tunable electric properties. PANI is easily prepared through oxidative polymerization of aniline [2]. Hybrids of PANI have been prepared by grafting it onto suitable inorganic bases such as clay or carbon-containing materials for applications [3–5], such as electromagnetic shielding, radar evasion, low-power rechargeable batteries, electronic devices, electrorheology, corrosion prevention, and sensors [6–8]. However, the absence of suitable solvents for doped PANI, its low mechanical properties, inability to be processed by conventional methods, and low thermal stability at high temperatures limit its practical applications. A variety of alternatives have been developed to address these limitations, including hybrid formation, blends, and composites of PANI.

Composites and blends of polymers containing conducting fillers have resolved many industrial problems [9–11]. Blending nanofillers into polymer composites facilitates the tailoring of composite properties. The electrical properties of conducting nanofillers and mechanical properties of a conventional polymer matrix are combined to synthesize a material with increased technological applications. Polymer composites are widely used in industrial applications due to their ability to combine the desired properties of various polymeric species. Composites of PANI with various polymers such as poly(vinyl chloride) (PVC), poly(vinyl acetylene), and polyethylene have been reported [12–14]. Among them, PVC offers more flexibility in mechanical properties ranging from elastomer to rigid plastic. It is one of the most frequently used materials in the creation of nanocomposites because of its comparatively low cost, well-established processing methods, modifiable mechanical properties, and its high environmental resistance. In a recent study, conducting polymers were grafted onto vinyl-modified sepiolite (VMS) to produce conducting nanohybrids (NHs) [15]. The PANI-grafted NH showed electrical conductivity of 4.27 × 10^−6^ S/cm· The electromagnetic interference (EMI) shielding effectiveness of the developed NH was also investigated in the X-band frequency range and found to be 24.2 dB. To explore the potential of this unique NH further, the current study combines this conducting nanofiller with a PVC matrix to produce NH polymer composites. Varying amounts of NH were incorporated into PVC by melt blending and their dielectric properties were examined using impedance spectroscopy (IS). IS is a useful technique to resolve conduction components by characterizing the transport properties of complex systems [16]. This technique is employed to characterize the electrical and dielectric properties in addition to charge transport mechanisms in conducting polymers. Structural, thermal, and mechanical properties of these NH polymer composites were also investigated.

## 2. Materials and methods

### 2.1. Materials

Commercial-grade PVC (grade: paste resin) from LG Chem, Korea, was used. Ba/Cd stearate (Goldstab 6140 one pack heat stabilizer, Goldstab Organics, India) and epoxidized soybean oil (99.9%) supplied by Hangzhou Dingyan Chemicals (Baiyang, China) were used as heat stabilizers. Commercial-grade dioctyl phthalate (DOP) (≥99.5%) was used as a plasticizer for PVC. All the chemicals were used without further purification. Sepiolite-grafted PANI NH was synthesized using a previously reported method [17]. Briefly, synthesis of PANI-grafted NH starts with the vinyl modification of sepiolite using vinyltriethoxysilane (VTES). The vinyl-modified sepiolite (MS) (1% w/v) was dispersed in demineralized water (DMW) in the presence of Tween 80 (3% w/v), followed by the addition of aniline (7% w/v) with constant stirring. Grafting was initiated by the dropwise addition of an aqueous solution of potassium persulfate (5% w/v) at 70 °C. Stirring was continued for 4 h at 70 °C, and the mixture was filtered and washed with HCl (5 M) to obtain sepiolite-grafted PANI conducting NH.

### 2.2. Preparation of the NH polymer composite

Varying amounts of nanofiller were mixed with PVC using the melt blending method in a HAAKE PolyLab Rheomix (Thermo Fisher Scientific, Karlsruhe, Germany). An appropriate amount of plasticizer, stabilizers, and NH was mixed with PVC in the internal mixer using roller rotors at 170 °C for 10 min at 80 rpm to prepare a series of PVC/NH composites [18]. Details of the compositions in terms of NH phr (parts by weight per hundred parts of PVC) are presented in Table 1. Stabilizer and plasticizer concentrations were kept constant in all formulations. The admixture was then hot pressed (Gibitre Instruments, Bergamo, Italy) at 150 °C and 100 bar for 3 min to fabricate 1 mm-thick sheets, followed by cold pressing to room temperature at 20 bar.

### 2.3. Characterization

A Fourier-transform infrared (FT-IR) spectrophotometer (Nicolet 6700, Thermo Electron Corporation, USA) was used for structural analysis of the NH composite samples. An average of 116 scans at 6 cm^−1^ resolution was recorded in the range of 4000–400 cm^−1^ using an ATR assembly. Thermal analysis was carried out using a TGA device from Mettler Toledo, Switzerland (Model: TGA/DSC1), at a heating rate of 10 °C/min from 50 to 600 °C under a 50 mL/min flow of nitrogen gas. Mechanical properties, including tensile strength and elongation at break, were investigated using a universal testing machine (SANS China) according to ASTM-D638 standard. Room temperature IS measurements were performed on NH polymer composites in a wide frequency range (0.1 Hz to 1 MHz) using an Alpha-N analyzer (Novocontrol, Montabaur, Germany). Electrical contacts were added to opposite sides of the samples using conductive silver ink, followed by curing under a tungsten lamp for 3 h. Leads were carefully checked to ensure the absence of any irrelevant resistive or capacitive coupling in the measured frequency range. To minimize the secondary effect of absorbed ambient moisture on the impedance of nanocomposites, all the measurements were taken on the same day by applying an AC signal of 1.0 V. Fully automated WinDETA software was used to interface the experimental setup of the analyzer with the computer and for data acquisition. Zview (Scribner Associates Inc., version 2.70) software was used for fitting the impedance data by keeping the reduced chi-squared (χ2) value at approximately 10^−5^ for best-fit equivalent circuits.

## 3. Results and discussion

### 3.1. FT-IR spectroscopy

**Figure 1:**
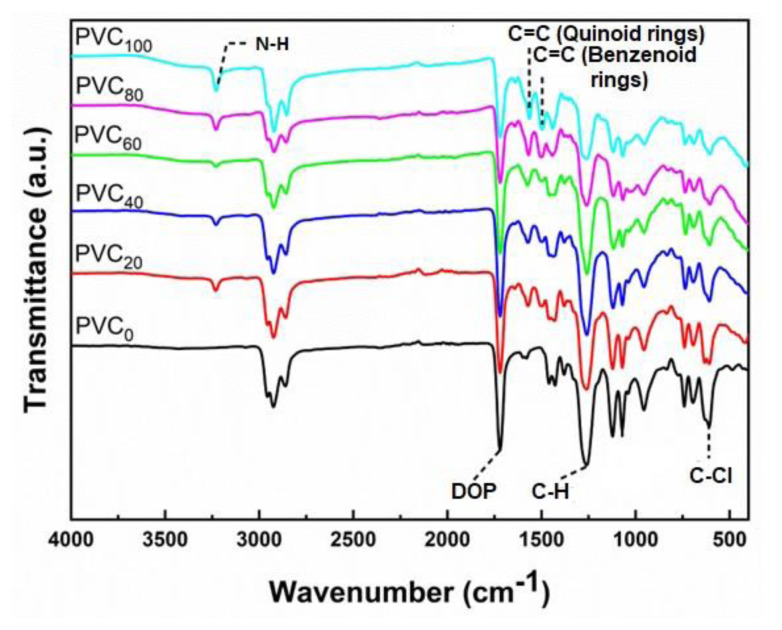
FT-IR spectra of NH polymer composites of PVC with variable NH contents.

The structural changes during composite formation were studied by FT-IR spectroscopy. Figure 1 shows the FT-IR spectra of PVC and its composites. The FT-IR spectrum of PVC_0_ shows the following characteristic peaks: C–H stretching vibrations appear in the region 2990–2830 cm^−1^, corresponding C–H bending vibrations are present at 1385 cm^−1^, and C–H rocking and trans C–H wagging appeared at 1263 cm^−1^ and 956 cm^−1^, respectively. A small kink at 1335 cm^−1^ corresponds to CH2 deformation. The peaks at 835, 745, 694, and 609 cm^−1^ are assigned to C–Cl stretching vibrations. Peaks at 1719, 1465, 1435, 1125, and 1072 cm^−1^ confirm the presence of the plasticizer (DOP).

The spectrum of PVC_20_ exhibits characteristic peaks of PVC as well as some additional peaks at 3232, 1568, and 1496 cm^−1^. The peak at 3232 cm^−1^ is assigned to the N–H stretching vibration. C=C stretching vibrations of quinoid and benzenoid rings appear at 1568 and 1496 cm^−1^, respectively. These peaks are observed in all the composites with different amounts of nanofiller. The increasing NH content in PVC decreases the peak intensities of characteristic PVC and DOP peaks. Peaks at 1573 cm^−1^ and 609 cm^−1^ were shifted to 1568 cm^−1^ and 603 cm^−1^, respectively, as the amount of nanofiller in PVC increased. These peak shifts may be due to the strong interactions between NH and the PVC matrix [19]. These results indicate that after the addition of NH, the chemical structure of the PVC matrix remains unchanged, facilitating the physical dispersion of NH filler into the PVC matrix.

### 3.2. Thermogravimetric analysis

**Figure 2:**
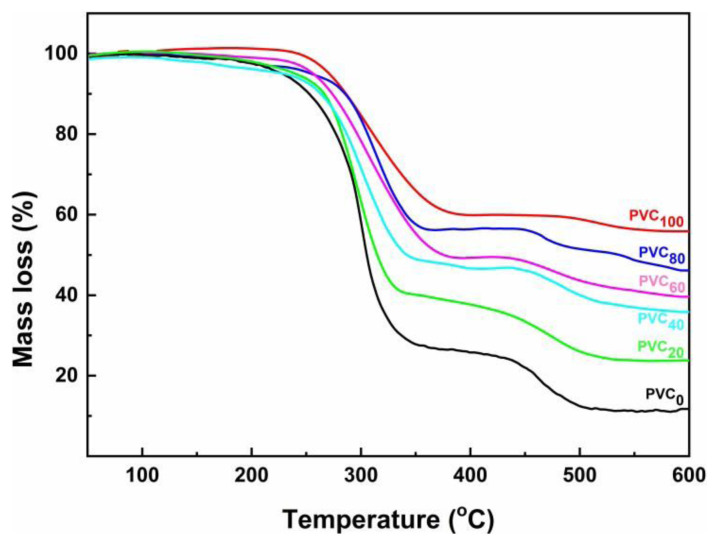
Thermograms of PVC_0_ and its NH composites as a function of NH content.

Thermograms of NH polymer composites prepared with different amounts of NH are presented in Figure 2. The onset decomposition temperature (T_o_), the fastest degradation temperature (T_fd_), the percentage of mass loss (M_loss_), and the residue (R) at 600 °C for each composite are summarized in Table 2. All the composites exhibit two-stage decomposition patterns. The first stage of decomposition between 200 °C and 390 °C may be attributed to the loss of volatiles including HCl. The onset decomposition temperature of PVC_0_ is around 200 °C, with a mass loss of 73.7% at 390 °C. Both the T_o_ and T_fd_ values of the nanocomposites are higher than those of PVC_0_. Moreover, it was also observed that increased NH content in the composite decreased the corresponding mass loss.

The second decomposition stage between 400 °C and 600 °C is due to the decomposition of the polymer backbone. The thermal stability of NH composites increases with the amount of NH due to the higher thermal stability of nanofiller particles [20–22].

### 3.3. Mechanical properties

**Figure 3:**
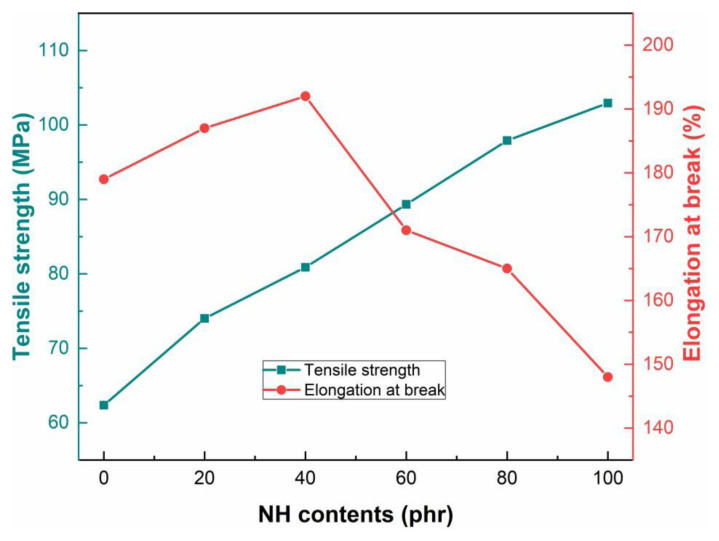
Effect of NH filler content on tensile strength and elongation at break of NH polymer composites.

The effect of NH content on mechanical properties (tensile strength and elongation at break) of PVC_0_ and PVC/NH composites was examined, and the results are presented in Figure 3. The plot shows that increasing NH content in the polymer matrix is positively correlated with tensile strength. Elongation at break initially increased up to 40 phr and then decreased as the NH content reached 100 phr. The initial increase in elongation at break can be attributed to the increased interaction between the nanofiller and polymer chains. The decrease in elongation at break at higher filler content is due to the increased filler-filler interaction that reduces the mobility of polymer chains [23].

### 3.4. Morphological analysis

**Figure 4:**
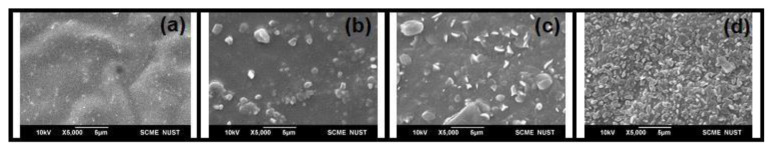
SEM micrographs of (a) PVC_0_, (b) PVC_20_, (c) PVC_40_, and (d) PVC_100_.

The surface morphology of the NH polymer composites was investigated using scanning electron microscopy (SEM), and SEM micrographs are presented in Figure 4. The surface morphology of the composites substantially differ depending on NH content. These topographical images show uniform mixing of the NH filler and a rugged surface morphology in all compositions. Ruggedness increases with increased concentration of NH.

### 3.5. IS analysis

**Figure 5:**
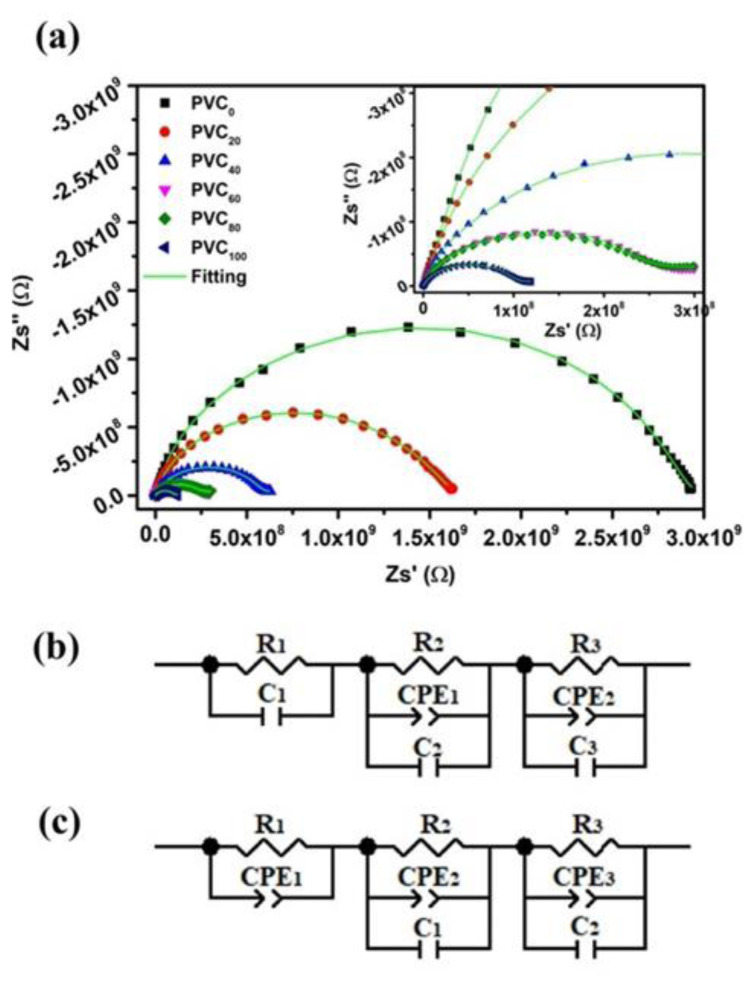
(a) Impedance plain plot of the synthesized NH composites where the solid line represents least square fitting to the data points. Fitted equivalent circuits for (b) PVC_0_ and (c) PVC/NH composites.

The effect of different concentrations of conducting nanofiller was examined using IS. Figure 5a shows the impedance plane plot along with least-squares fitting for PVC_0_ and the NH composites. It is evident from the figure that the increased nanofiller loading introduced conducting channels, thereby enhancing charge carrier transport within the composites and decreasing the impedance. However, the decrease in impedance abruptly halts after PVC_60_ and then resumes upon further increase in the nanofiller content up to 100 phr (inset of Figure 5a). This behavior might be due to sudden changes in the conduction mechanism occurring after 60 phr loading. A collection of typical impedance datasets reveals similar features of a depressed semicircular arc whose width is larger than its height ( $Z_{max}^{\prime \prime }<\frac{1}{2}[Z_{max}^{\prime }-Z_{min}^{\prime }]$), where $Z_{max}^{\prime }$ and $Z_{min}^{\prime }$ are the intercepts of the arc with the real axis at low and high frequencies, respectively. The presence of defects, structural stresses, and distributed components of the matrix displaces the center of the impedance semicircle below the real axis. The constant phase element (CPE) is used to address the heterogeneity of the samples instead of a capacitor, to account for the nonideal behavior (i.e. depression of semicircular arc center below the Z′-axis) in order to fit the impedance plan plot. The CPE can be deconvoluted into resistive and capacitive components, whose relative contribution is given by the CPE parameter *n*, which is a measure of deviation from the ideal behavior. For pure resistive behavior, its value is zero and for capacitive behavior, it has a unity value. The capacitance of a CPE is given as [24]:


C=R(1-n)/n (CPE)1/n

Here, C and R are capacitance and resistance, respectively, of the related component.

Figures 5b and 5c show the fitted equivalent circuits for PVC_0_ and PVC/NH composites, respectively. Fitted parameters for all components of equivalent electrical circuits are given in Table 3. The fitted circuits consist of three distinct sections, two of which remain virtually unchanged for PVC_0_ and PVC/NH composites. The first section comprises a resistor and a capacitor joined in parallel. In the case of PVC/NH composites, the capacitor is replaced with a CPE, indicating the introduction of heterogeneous capacitance in the system. This conclusion is supported by the fitted parameter *n* in Table 3, which decreases with increasing nanofiller loading. The apparent behavior in the impedance plain plot is complicated to match the fitted equivalent circuits to despite the good fitting characteristics and therefore requires individual analysis of conductivity, imaginary part of impedance, and electrical modulus.

**Figure 6:**
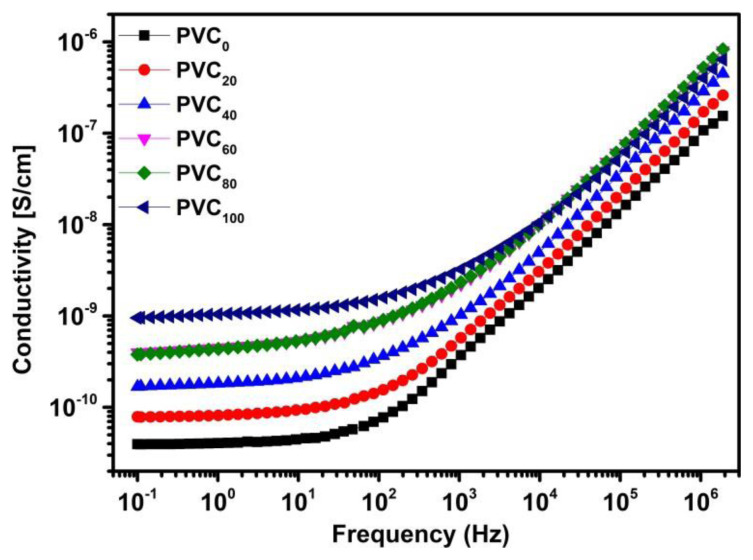
Frequency dependent conductivity of PVC/NH composites.

Figure 6 shows the frequency-dependent conductivity response of the samples. The conductivity at low frequencies is fairly constant and representative of DC conductivity, while a sharp rise at higher frequencies is typical behavior for conducting polymers. The results complement the findings from the impedance plane plot, where the increased amount of NH in the composite increases conductivity. It stalls after 60 phr but increases again up to 100 phr at lower frequencies. This behavior is also observed at higher frequencies, where conductivity of PVC_100_ is lower than that of PVC_60_. These complementary results strengthen the conclusion regarding a change in conduction mechanism above 60 phr loading.

**Figure 7:**
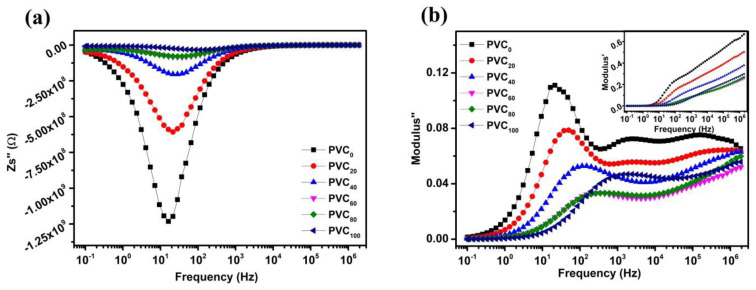
Frequency response of imaginary parts of (a) impedance and (b) electric modulus for PVC/NH composites.

Generally, together with impedance, modulus formalism is used to separate microscopic processes responsible for localized and long-range conduction. Plotting Z″ and M″ against frequency shows if the resistance in PVC_0_, as shown by the largest arc in plots of Z″ vs Z′, is localized or widespread. Figure 7a plots the imaginary part of impedance against frequency, showing the main relaxation in the samples, which is suppressed by the loading of NH. The relaxation frequency tends to shift gradually towards the higher frequency range. Figure 7b shows the variation of the imaginary part of the electric modulus (M″), which further resolves the relaxations seen in Figure 7a into three distinct peaks in the frequency landscape, where peak positions closely resemble the values calculated by the fitted models. The real part of electric modulus (M′) (inset of Figure 7b) approaches zero at low frequencies, indicating a minuscule contribution from the contact, as discussed by Lee et al. [25]. However, in this case, the samples have several constituents in varying concentrations as described in Table 1.

Collectively, the findings suggest that the relaxations in Figure 7b arise from the intrinsic response of the PVC/NH composites and added components, i.e. the plasticizer and stabilizers, at different frequency ranges. This figure also shows that increase in NH content in the composites results in the suppression of low-frequency relaxation up to 80 phr (PVC_80_). Such behavior might arise from the increased networking of NH and its interaction with polarized chlorine atoms (Cl^δ−^) in PVC chains. It is worth mentioning that the low-frequency relaxation peak is a composite peak formed by merging adjacent relaxations that tend to broaden with the increasing NH content. This suggests multiple components, such as intrinsic PVC response, NH response, a combination of hydrogen bonding, and possible van der Waals interactions, are at play. As a result, more charge carriers are trapped, which were previously travelling through conjugation on the polymer backbone. This suppressive behavior is reversed above 60 phr loading, and the relaxation reappears at a higher frequency, which might result from a change in the transport conduction mechanism. The high-frequency relaxation due to additives is similarly suppressed. However, the variation is lower than that for the low-frequency relaxation. Similar to the first relaxation, a reversal of the effect is also partially visible after 60 phr (PVC_60_). Additionally, this relaxation also tends to shift towards the higher frequency range. Intermediate relaxation due to a fixed concentration of additives is suppressed by increasing NH content. The peak also shifts towards the lower frequency range and merges with the low-frequency relaxation, which shifts towards higher frequencies.

**Figure 8:**
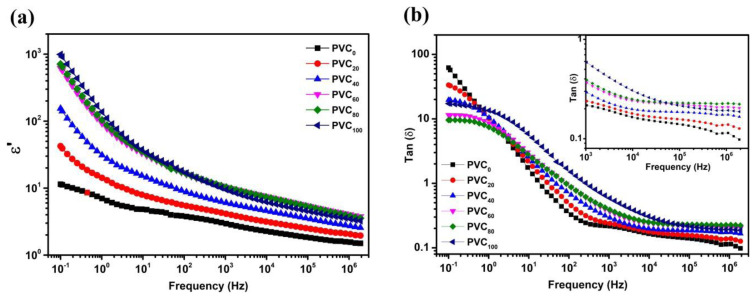
(a) Variation of dielectric constant and (b) tangent loss spectrum for PVC/NH composites as a function of frequency.

Figure 8a shows the variation of dielectric constant with frequency. The dielectric constant is fairly stable for PVC_0_ but exhibits increasingly degraded behavior with increasing frequency and NH content in composite samples. Although NH loading greatly increases the dielectric constant at lower frequencies, this is typical for PANI. The variation at high frequencies is, however, not that prominent. The dielectric loss (tanδ) spectrum is also stable at high frequencies, as shown in Figure 8b. The loss spectrum can be explained by the dipolar relaxations from conjugation on the polymeric backbone. The increase in NH content slightly increases the dielectric loss while pushing the onset of stability towards higher frequencies. This suggests a higher energy requirement for polarization switching, thereby confirming network formation via hydrogen bonding. Such behavior is consistent with the overall conclusions about the electrical properties.

## 4. Conclusion

PVC/NH composites were prepared by melt blending varying amounts of NH into PVC. Strong interactions between the individual components of NH composites are reflected in FT-IR spectroscopy. Uniform nanofiller dispersion and enhanced interfacial bonding between the individual components of the composite resulted in improved thermal stability and mechanical properties. Maximum elongation at break of 192% was observed in 40 phr. Complex impedance analysis revealed the electrical transport properties of PVC/NH nanocomposites at room temperature. High dielectric constant values at low frequencies are attributed to increased interfacial interaction in the nanocomposite. Enhancement in conductivity of the nanocomposite from 0.6 × 10^−7^ S/cm^−1^ to 7.9 × 10^−7^ S/cm^−1^ is observed with the increase in conducting nanofiller content. The substantial improvement in the electrical properties of nanocomposites with increased NH concentration indicates their potential use as conducting nanofillers in future polyblends. This could be beneficial for a variety of applications in the field of biomedicine, biosensing, electromagnetic interference shielding, and environmental sensing.

## Figures and Tables

**Table 1:** Codes and composition of the NH polymer composites formulations. Ba-Cd stearate = 3 phr, epoxide oil = 1.5 phr, DOP = 60 phr.

**Code**	PVC_0_	PVC_20_	PVC_40_	PVC_60_	PVC_80_	PVC_100_
**NH (phr)**	0	20	40	60	80	100

**Table 2:** Onset temperature, fastest degradation temperature, mass loss at different degradation stages and residual mass of PVCo and PVC/NH composites.

	1st stage			2nd stage			R (%)
Sample (°C)	T_0_	T_fd_ (°C)	M_loss_ (%)	T_0_ (°C)	T_fd_ (°C)	M_loss_ (%)	at 600 °C
PVC_0_	200	295	73.7	400	463	6.9	11.2
PVC_20_	220	300	62.0	405	474	14.3	23.7
PVC_40_	220	303	53.3	427	480	10.8	35.9
PVC_60_	230	308	50.5	436	484	10.0	39.5
PVC_80_	240	312	44.7	446	490	9.1	46.2
PVC_100_	250	315	40.1	458	520	5.4	54.5

**Table 3:** Fitting parameters for equivalent circuit fitted for PVCo and PVC/NH composites.

	PVC_0_	PVC_20_	PVC_40_	PVC_60_	PVC_80_	PVC_100_
**R** **_1_**	2.5145×10^8^	7.3263×10^7^	2.9793×10^8^	1.0879×10^8^	1.2855×10^8^	5.2897×10^7^
**CPE** **_1_**	1.3728×10^−14^	2.042×10^−11^	1.4776×10^−11^	1.9463×10^−11^	1.7711×10^−11^	1.6477×10^−11^
**n** **_1_**	0.28071	0.9914	0.9006	0.855	0.8366	0.8397
**R** **_2_**	2.7791×10^9^	1.5676×10^9^	4.942×10^8^	4.745×10^8^	7.6289×10^8^	1.2279×10^8^
**CPE** **_2_**	6.455×10^−12^	8.5856×10^−13^	1.8226×10^−9^	2.1629×10^−7^	2.1953×10^−5^	5.3729×10^−8^
**n** **_2_**	0.73293	0.4794	0.1389	0.1354	0.1398	0.1414
**C** **_1_**	1.0066×10^−11^	4.1738×10^−12^	6.1953×10^−11^	6.6891×10^−11^	9.2369×10^−11^	8.9272×10^−11^
**R** **_3_**	4.0827×10^7^	2.4005×10^7^	6.9434×10^7^	2.0861×10^7^	9.4349×10^6^	1.1451×10^7^
**CPE** **_3_**	–	9.0537×10^−12^	1.4099×10^−11^	2.776×10^−11^	2.4248×10^−11^	1.4271×10^−11^
**n** **_3_**	–	0.7315	0.8239	0.7985	0.7341	0.6248
**C** **_2_**	4.1272×10^−12^	1.15×10^−12^	1.3581×10^−12^	3.8932×10^−12^	1.7389×10^−11^	7.7525×10^−12^
**C** **_3_**	1.114×10^−12^	–	–	–	–	–
**χ** **^2^**	1.32 ×10^−4^	6.464×10^−5^	8.708×10^−5^	5.1956×10^−5^	1.74×10^−5^	6.66×10^−5^
**Sum of squares**	0.012727	0.0066579	0.00984	0.0058711	0.001967	0.007526
